# Novel Biopsy Gun Impacts Biopsy Sample Quality and Quantity

**DOI:** 10.1007/s00270-025-04162-z

**Published:** 2025-08-20

**Authors:** Laetitia Saccenti, Tabea Borde, Nicole A. Varble, Lindsey Hazen, Ming Li, Michael Kassin, Ifechi Ukeh, Sandeep Gurram, Peter A. Pinto, Ivane Bakhutashvili, William F. Pritchard, John W. Karanian, Bradford J. Wood

**Affiliations:** 1 Center for Interventional Oncology, Radiology and Imaging Sciences, Clinical Center, National Institutes of Health, Bethesda, MD 20892 USA; 2 Henri Mondor Biomedical Research Institute, Inserm U955, Team No 18, Créteil, France; 3 Philips Healthcare, Cambridge, MA 02141 USA; 4 Urologic Oncology Branch, National Cancer Institute, National Institutes of Health, Bethesda, MD USA; 5 National Institute of Biomedical Imaging and Bioengineering, Bethesda, MD 20892 USA

**Keywords:** Biopsy, Large-core needle, Equipment design, Precision medicine, Interventional radiology

## Abstract

**Purpose:**

A biopsy gun featuring alternated serrated cutting edges was designed to improve core stability and tissue acquisition. This study aimed to assess the impact upon core biopsy tissue quantity and quality of a serrated core gun (SUREcore prime, Uro-1).

**Materials and methods:**

18G serrated core gun was compared with 18G conventional gun (Maxcore, Becton Dickinson), in both ex vivo (bovine liver, N = 30) and in vivo (swine liver and kidney, N = 24) models. Cores were assessed for weight, length, surface area, fragmentation, and solidity using digital pathology.

**Results:**

The serrated core gun produced cores with 16% higher median weight (6.60 g vs. 5.70 g, *p* < 0.001), 10% longer length (11.1 mm vs. 10.1 mm, *p = *0.042), and 16% greater surface area (7.80mm^2^ vs. 6.70mm^2^, *p = *0.024). Fragmentation and solidity did not differ significantly. Subgroup analyses confirmed higher tissue weight across all organs and test conditions. No hemorrhagic complication was observed on post-procedural CT or autopsy.

**Conclusion:**

The serrated-edge design may improve biopsy sample quality and quantity without increasing needle size or penetration depth, potentially reducing the need for repeated passes. Device research and innovations may further improve biopsy efficacy and outcomes and reduce complications.

**Graphical Abstract:**

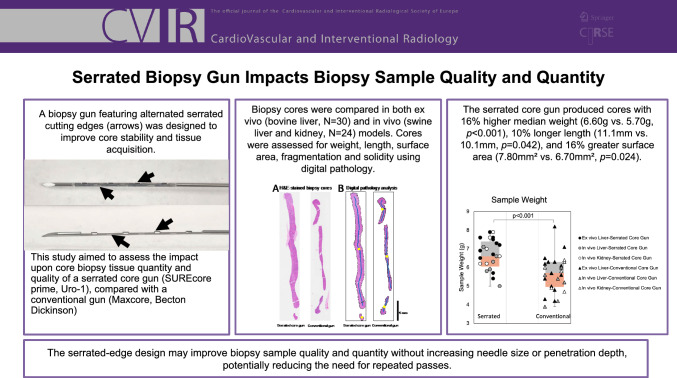

## Introduction

Modern designs for percutaneous core needle biopsy devices, including widely available side-cutting needles, were introduced in the 1960s [1], with few improvements made since. Larger core enhances diagnostic value [2, 3], especially in the era of personalized medicine, which relies upon molecular [4] and genetic testing [5]. If tissue quality or quantity is inadequate, additional passes may be indicated. Similarly, a larger gauge needle may increase risk of complications depending on tissue type [6, 7].

This study aimed to evaluate whether a new biopsy device with custom cutting edges improves biopsy core quality in terms of weight, surface area, and fragmentation in different organs in an ex vivo and in vivo model.

## Material and Methods

### Biopsy Devices

**Fig. 1:**
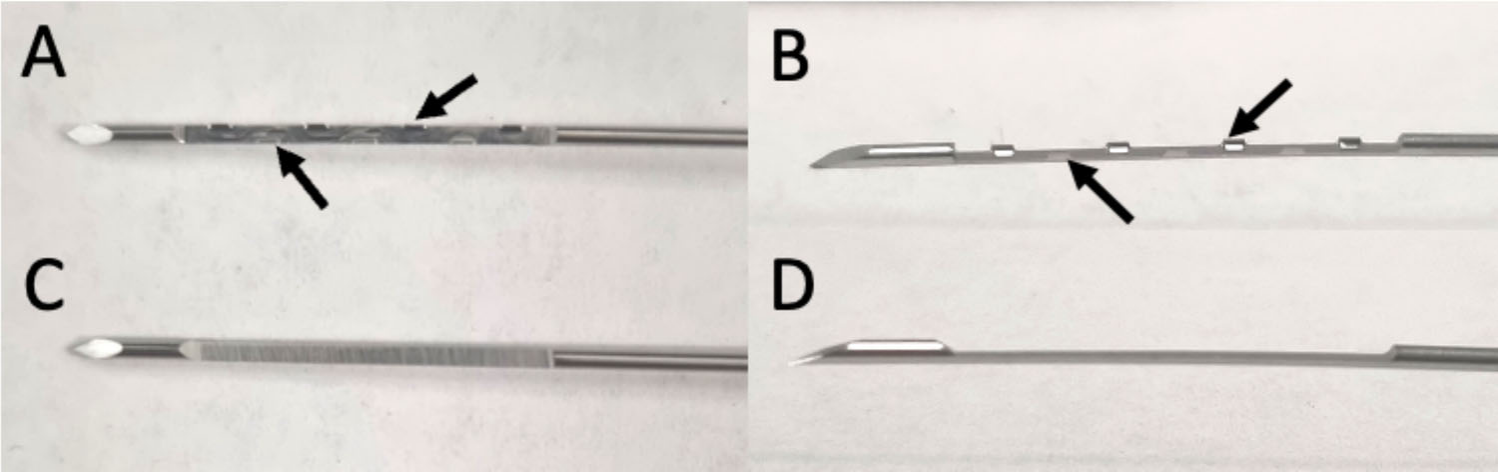
Comparison of biopsy gun devices. Front (**A**) and profile (**B**) of the serrated core gun with serrated cutting edges (arrows). Front (**C**) and profile (**D**) of the conventional biopsy gun. Both devices have similar penetrating length and similar outer diameter but different sample notch designs

**Fig. 2:**
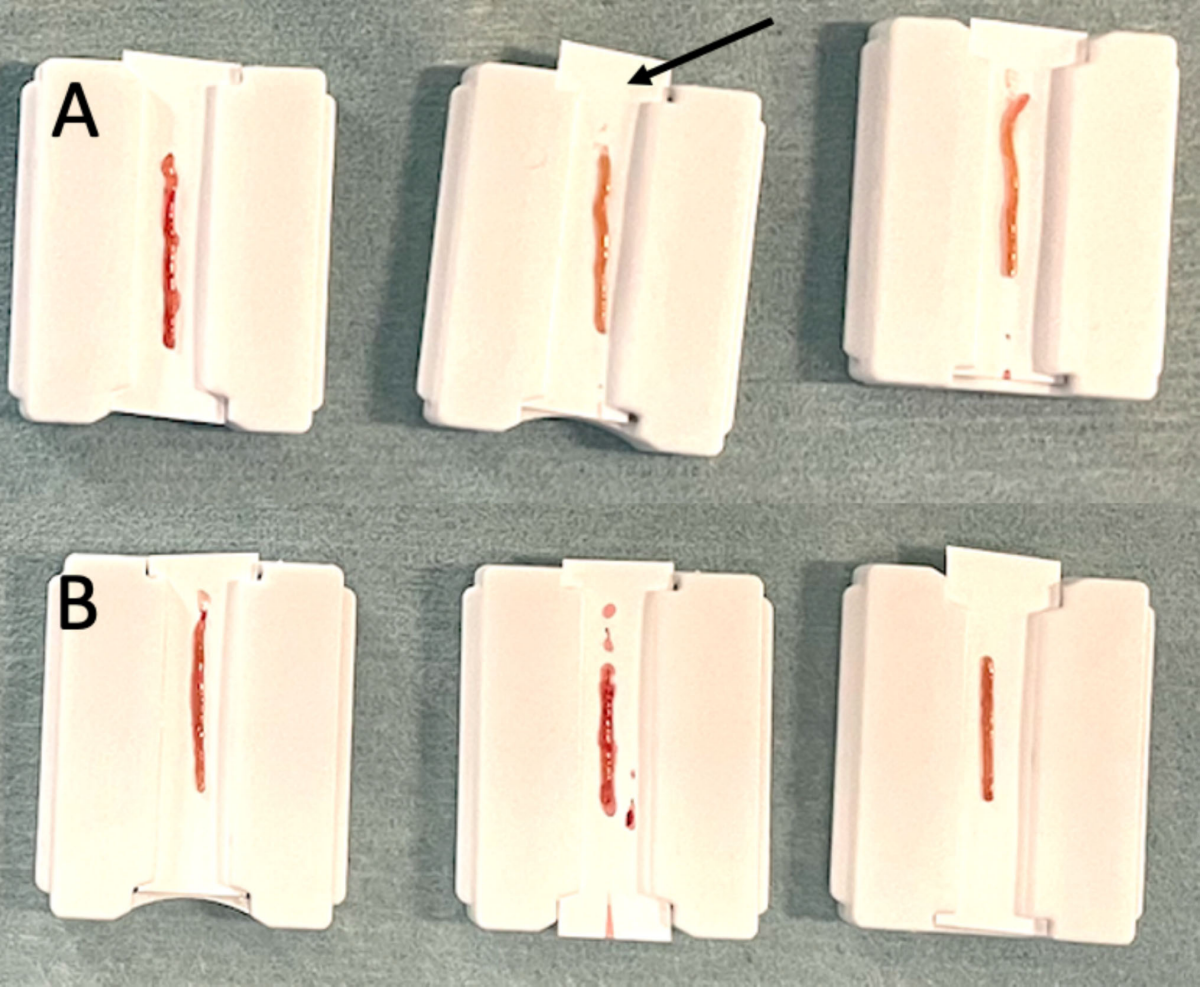
Examples of kidney biopsy cores performed with the (**A**) new custom biopsy gun and (**B**) standard of care conventional gun. Each sample was transferred on a paper holder (arrow) designed to help with core transfer thanks to electrostatic forces. The samples of the conventional gun were thinner (B left), more fragmented (B middle), or shorter (B right) than the ones from the serrated biopsy gun

A biopsy gun was custom-designed and fabricated with serrated cutting edges (Fig. 1) (SUREcore prime, URO-1 Medical, Greensboro, USA). These cutting edges were alternately arrayed laterally on each side of the sample notch, engineered to stabilize the tissue and minimize motion during acquisition. The study device 18-gauge, 20-cm serrated core gun had a 19 mm sample notch, 22 mm penetration depth, and 3 mm dead space. It was compared to a conventional 18-gauge, 25-cm core gun with a 18-mm sample notch, 22 mm penetration depth, and 4 mm dead space (Maxcore, Becton Dickinson, Franklin Lakes, USA) (Fig. 1). To avoid bias and focus on the difference in biopsy device design, all cores were removed from the biopsy devices using a positively charged tissue transfer “coreCARE” (URO-1 Medical, Greensboro, USA, Fig. 2).

### Ex Vivo Evaluation

Three physicians (one resident, one fellow, and one senior interventional radiologist) performed a total of 30 biopsies of a fresh bovine liver using both the serrated core gun and the conventional gun (N = 15 per device). Biopsies were performed under ultrasound guidance to avoid vessels and fascia. Each core was extracted, weighed, and photographed, and then fixed with formalin. Cores were then paraffin embedded and stained with hematoxylin and eosin (H&E).

### In Vivo Evaluation

A male Yorkshire domestic swine was studied under a protocol approved by the Institutional Animal Care and Use Committee. The subject was intubated under general anesthesia and mechanically ventilated.

Two interventional radiologists (one resident and one fellow) performed a total of 12 biopsies in liver and 12 biopsies in kidney, using both the serrated core gun and the conventional gun (*N* = 6 liver biopsies per device and *N* = 6 kidney biopsies per device) under ultrasound guidance. Each biopsy core was extracted, weighed, and photographed, and then fixed with formalin. Cores were paraffin embedded and stained with H&E. Safety of the biopsies was assessed on an unenhanced abdominal CT scan (iQon, Philips, Best, Netherlands) and with post-mortem analyses.

### Biopsy Quality Analysis

Automatic measurements of core length, surface area, solidity (quantification of irregularity of the shape), and number of fragments were performed on digital pathology (MATLAB, R2023a). The number of renal glomeruli was quantified by one physician.

### Statistics

The normality of data was assessed using the Shapiro–Wilk test. All measurements were expressed as median (interquartile range). Results were compared using the Wilcoxon rank sum test and Fisher’s exact test. Statistical significance was set at p < 0.05. Data were analyzed using Rstudio (version 2024.12.1 + 563). All cohorts were analyzed separately and pooled and analyzed by needle type.

## Results

### Biopsy Quality Data

**Fig. 3:**
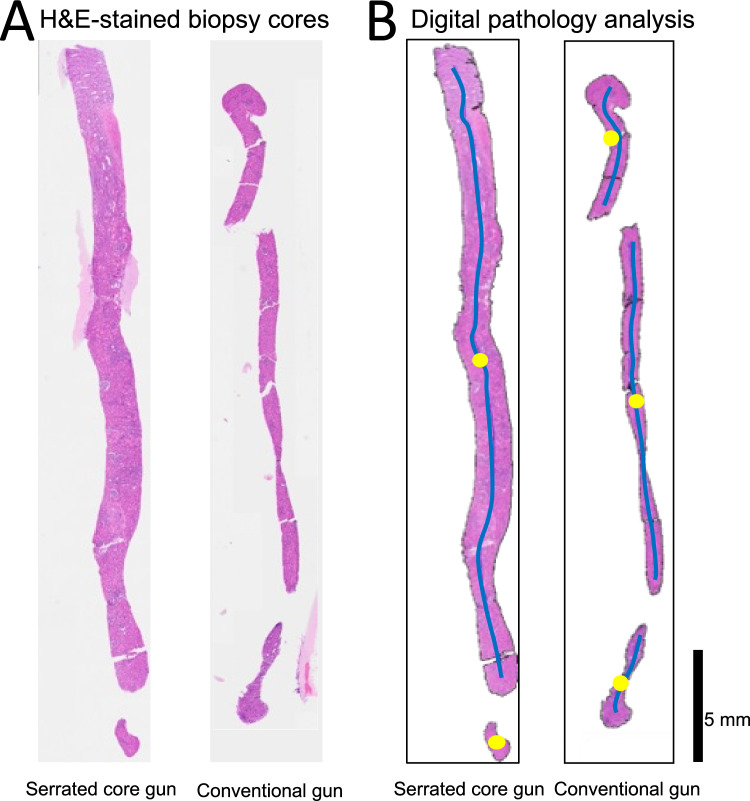
**A** Representative digitized hematoxylin and eosin (H&E)-stained kidney biopsy core examples for the serrated core gun (left) and the conventional gun (right). **B** Segmented and automatically processed digitized images. The blue line is the extracted centerline from which the biopsy core length was calculated. The yellow dot shows the centroid of each fragment. This specimen from the serrated core gun has less artifact and less irregularity at the edge than the specimen from the conventional gun

**Fig. 4:**
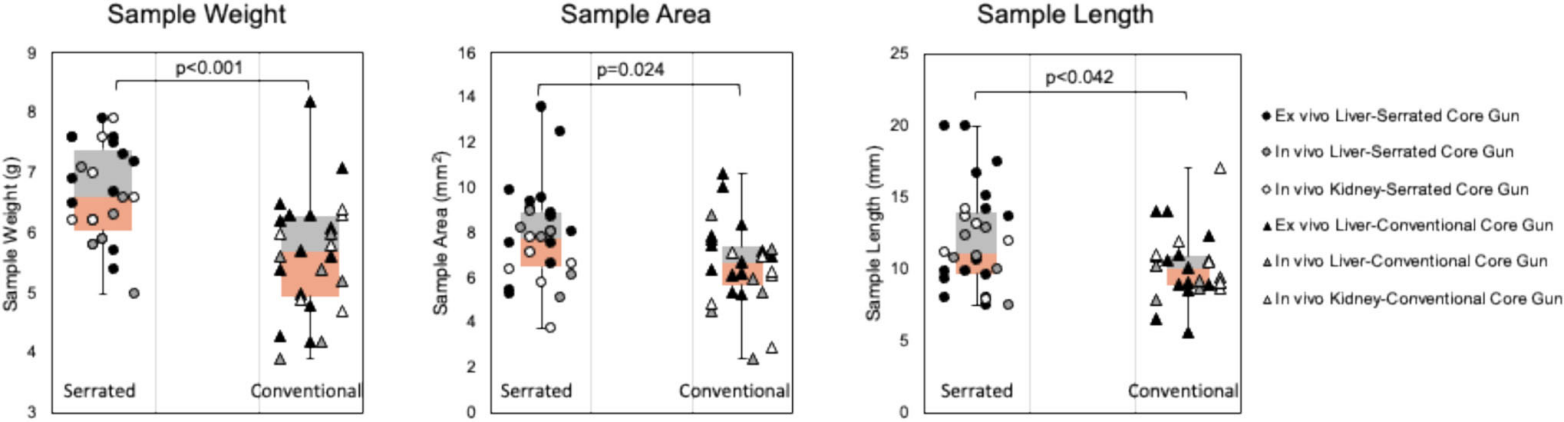
Comparison of sample weight, area, and length between the serrated core gun and the conventional core gun. The samples obtained with the serrated core gun tended to be heavier and larger than the ones obtained with the conventional core gun, whichever their original cohort (ex vivo versus in vivo, kidney versus liver)

A total of fifty-four biopsy cores were analyzed (27 cores/device, Figs. 3 and 4, Table 1). For pooled ex vivo and in vivo samples, the median weight of the biopsy cores using the serrated core gun was 16% higher than the median weight using the conventional gun (6.60 g [6.05–7.40]; versus 5.70 g [4.95–6.30], *p* < 0.001, Fig. 1c). There was no visible imbalance of blood macroscopically between the biopsy devices.

**Table 1:** Comparison of biopsy cores obtained from serrated core gun and conventional gun. All cores from ex vivo and in vivo experiments were pooled together

Characteristic	Serrated core gun (*n* = 27)	Conventional gun (*n* = 27)	*p*-value
Weight (g)	6.60 (6.05–7.40)	5.70 (4.95–6.30)	< 0.001
Total area (mm^2^)	7.80 (6.50–8.95)	6.70 (5.7–7.40)	0.024
Area of largest fragment (mm^2^)	5.50 (4.80–7.25)	4.80 (3.55–7.05)	0.11
Solidity	0.67 (0.55-0.79)	0.74 (0.66–0.80)	0.13
Solidity of largest fragment	0.54 (0.41–0.68)	0.66 (0.56–0.72)	0.047
Total length (mm)	11.10 (9.70–13.95)	10.10 (8.90–10.95)	0.042
Length of largest fragment (mm)	8.0 (6.2–11.2)	7.6 (5.5–9.5)	0.2
Number of fragments	3.0 (2.0–4.0)	3.0 (1.5–4)	0.55

Data are expressed as median (interquartile range).

In digital pathology, the biopsy core length using the serrated core gun was 10% larger than with the conventional gun (11.1 mm [9.70–13.95] versus 10.1 mm [8.90–10.95]; *p = *0.042). The surface area of the biopsy core using the serrated core gun was 16% larger than with the conventional gun (7.80mm^2^ [6.50–8.95] versus 6.70mm^2^ [5.70–7.40]; *p = *0.024, Table 1). The number of fragments, solidity, and number of glomeruli were similar between the two groups (respectively, *p = *0.55, *p = *0.13, and *p = *0.30).

### Subgroups Analysis

Ex vivo and in vivo, as well as liver and kidney subgroups analyzed independently, showed that weight was higher using the serrated core gun compared to the conventional gun. All other parameters were similar (Tables 2 and 3).

**Table 2:** Comparison of in vivo liver biopsy cores obtained from serrated core gun and conventional gun

Characteristic	Serrated core gun (*n* = 6)	Conventional gun (*n* = 6)	*p*-value
Weight (g)	6.10 (5.83–6.53)	5.30 (4.45–5.55)	0.065
Total area (mm^2^)	7.95 (6.53–8.18)	5.70 (4.73–6.98)	0.2
Area of largest fragment (mm^2^)	5.10 (4.45–5.90)	5.50 (4.73–6.88)	0.8
Solidity	0.71 (0.61–0.78)	0.66 (0.63–0.72)	> 0.9
Solidity of largest fragment	0.67 (0.61–0.79)	0.65 (0.58–0.68)	0.8
Total length (mm)	10.85 (10.20–12.03)	9.30 (8.75–10.08)	0.13
Number of fragments	1.5 (1.0–2.75)	3.5 (1.25–5.0)	0.89
Length of largest fragment (mm)	6.70 (6.20–7.43)	8.65 (7.68–9.55)	0.3

Data are expressed as median (interquartile range).

**Table 3:** Comparison of in vivo kidney biopsy cores obtained from serrated core gun and conventional gun

Characteristic	Serrated core gun (*n* = 6)	Conventional gun (*n* = 6)	*p*-value
Weight (g)	6.80 (6.30–7.45)	5.90 (5.13–6.23)	0.030
Total area (mm^2^)	6.50 (5.95–6.98)	6.20 (5.20–6.83)	0.5
Area of largest fragment (mm^2^)	6.45 (5.35–6.95)	3.85 (3.08–6.28)	0.2
Solidity	0.71 (0.48–0.79)	0.69 (0.66–0.76)	0.8
Solidity of largest fragment	0.52 (0.41–0.65)	0.66 (0.57–0.77)	0.13
Total length (mm)	12.50 (11.30–13.55)	10.75 (9.38–11.68)	0.4
Number of fragments	2.0 (1.25–2.75)	2.0 (1.25–2.0)	0.31
Length of largest fragment (mm)	11.20 (10.50–13.10)	9.05 (6.65–10.18)	0.13
Number of glomeruli	15.0 (11.3–18.8)	9.0 (8.0–12.3)	0.3

Data are expressed as median (interquartile range).

Median weight of the ex vivo biopsy cores (N = 15/device) using the serrated core gun (6.90 g [6.05–7.55]) was higher than median weight using the conventional gun (6.10 g [5.20–6.30], *p = *0.018). The median weight of the in vivo biopsy cores (N = 12/device) using the serrated core gun (6.45 g, [6.13–7.03]) was higher than using the conventional gun (5.50 g [4.85–6.00], *p = *0.004). The median weight of the biopsy cores in the kidney (N = 6/device) using the serrated core gun (6.80 g [6.30–7.45]) was higher than using the conventional gun (5.90 g [5.13–6.23], *p = *0.030). All other pathology results for ex vivo and in vivo results, and organ-specific sub-analyses were similar (Tables 2 and 3).

Post-procedural CT showed no identifiable hemorrhage.

## Discussion

An improved efficacy of tissue acquisition may impact patient care. Enhancing the design of biopsy guns led to an increase in tissue weight and surface area by 16%. It is possible that adding alternated serrated cutting edges at the lateral sides of the sample notch may stabilize the positioning of the biopsy core without pushing the tissue away while deploying the inner edge of the biopsy device. The hypothesis behind the design was that the serrated edges would hold in place the tissue and limit tissue motion and migration that might otherwise result in fragmentation or alter the length or width of the biopsy core compared to standard flat notch design. Although the results about fragmentation were not conclusive in this study, biopsy quality was increased in terms of tissue quantity.

Biopsy core quality enhances diagnostic accuracy. Biopsies of liver lesions have a reported diagnostic accuracy of 83% and 1 in 5 cores may be inadequate [8, 9]. Moreover, advances in cancer diagnosis and personalized management often mandate higher quantities of pathologic tissue for genomic profiling and characterization allowing for more targeted treatment strategies [10, 11]. Research biopsy has also become a foundational technology in early phase clinical trials for drug discovery [12]. In this study, biopsy cores with more tissue were obtained using the same penetrating length and diameter of the biopsy gun. This biopsy gun design could enhance diagnostic value without increasing the risk of complications.

This study has limitations. Biopsy core weight may be influenced by the presence of blood in the core. Fragmentation may have increased while transferring the biopsy core to the cassette and during processing of the tissue in histopathology; however, all tissue cores were treated equally between biopsy devices. One theoretical confounding factor might have been the effect of multiple samples and the statistical pooling of cohorts along gun type and ex vivo and in vivo. Finally, the analysis included a limited number of biopsies, and more data are required to verify the safety of the device.

## Conclusion

A biopsy gun with serrated cutting edges may increase the volume of the biopsy core without modifying the physical penetrating length and diameter. Such device innovations provide the foundation for further clinical study of practical utility or impact upon biopsy efficacy and outcomes. With a serrated biopsy gun design, there is a direct link between biopsy gun cutting mechanism and the quantity of core specimen in both kidney and liver. Methods for enhancing biopsy quantity and quality play critical and expanding roles in the era of personalized molecular therapeutics.

## Declarations

### Human and Participants

This article does not contain any studies with human participants performed by any of the authors.
